# Drug responses are conserved across patient-derived xenograft models of melanoma leading to identification of novel drug combination therapies

**DOI:** 10.1038/s41416-019-0696-y

**Published:** 2019-12-20

**Authors:** Ryan J. Ice, Michelle Chen, Max Sidorov, Tam Le Ho, Rinette W. L. Woo, Aida Rodriguez-Brotons, Tri Luu, Damon Jian, Kevin B. Kim, Stanley P. Leong, HanKyul Kim, Angela Kim, Des Stone, Ari Nazarian, Alyssia Oh, Gregory J. Tranah, Mehdi Nosrati, David de Semir, Altaf A. Dar, Stephen Chang, Pierre-Yves Desprez, Mohammed Kashani-Sabet, Liliana Soroceanu, Sean D. McAllister

**Affiliations:** 1 California Pacific Medical Center Research Institute, San Francisco, CA 94107 USA; 2 University of California at San Francisco, School of Pharmacy, Department of Clinical Pharmacy, San Francisco, CA 94143 USA

**Keywords:** Melanoma, Cancer prevention

## Abstract

**Background:**

Patient-derived xenograft (PDX) mouse tumour models can predict response to therapy in patients. Predictions made from PDX cultures (PDXC) would allow for more rapid and comprehensive evaluation of potential treatment options for patients, including drug combinations.

**Methods:**

We developed a PDX library of BRAF-mutant metastatic melanoma, and a high-throughput drug-screening (HTDS) platform utilising clinically relevant drug exposures. We then evaluated 34 antitumor agents across eight melanoma PDXCs, compared drug response to BRAF and MEK inhibitors alone or in combination with PDXC and the corresponding PDX, and investigated novel drug combinations targeting BRAF inhibitor-resistant melanoma.

**Results:**

The concordance of cancer-driving mutations across patient, matched PDX and subsequent PDX generations increases as variant allele frequency (VAF) increases. There was a high correlation in the magnitude of response to BRAF and MEK inhibitors between PDXCs and corresponding PDXs. PDXCs and corresponding PDXs from metastatic melanoma patients that progressed on standard-of-care therapy demonstrated similar resistance patterns to BRAF and MEK inhibitor therapy. Importantly, HTDS identified novel drug combinations to target BRAF-resistant melanoma.

**Conclusions:**

The biological consistency observed between PDXCs and PDXs suggests that PDXCs may allow for a rapid and comprehensive identification of treatments for aggressive cancers, including combination therapies.

## Background

Effective therapies have been developed to treat a subset of metastatic melanoma, including the use of kinase inhibitors (BRAF and MEK) and immune checkpoint blockade.^1^ Melanoma, however, has a high mutational burden,^2^ and a significant proportion of cases adapt to current therapies and progress. As a result, additional treatment strategies are needed for patients with cancers resistant to the standard-of-care.^3^ In the development of effective personalised therapy platforms for cancer patients, research has focused on the use of patient-derived xenografts (PDXs). In a recent study, researchers compared drug response of 92 patients with response observed in their corresponding PDX model.^4^ Their analysis showed that the response in PDXs predicted the response in the patient 87% of the time, even as patients underwent several cycles of therapy. This is in agreement with other studies demonstrating the utility of PDXs to predict clinical responses.^5–8^

Development of PDX technology to make precision medicine predictions for patients requires significant financial investment, time for tumour engraftment and serial passaging (2–8 months). Most importantly, it is not amenable to the rapid identification of multiple single drug candidates, or drug combinations for targeting cancer, the latter being critical for addressing advanced cancers resistant to single agents. By contrast, PDXCs used in a HTDS format represent a high speed and lower cost opportunity to study the activity of multiple single agents and drug combinations. Prior studies have shown that a robust drug response in PDXCs leads to some measure of significant activity in vivo in the corresponding PDXs.^9,10^ However, detailed studies demonstrating a direct statistical correlation in the magnitude of targeted therapy drug response between PDXCs and corresponding PDXs are needed. The existence of a strong correlation would increase support for the utility of PDXCs as a model for discovery of drug combination therapies targeting resistant cancers.

In this study, we first developed a PDX library of BRAF-mutant metastatic melanoma, with accompanying DNA mutational profiling of key driver genes, as well as clinical annotation. Using this library, we then assessed the utility of PDXCs, and a HTDS platform optimised for patient-derived tumour models and utilising clinically relevant drug exposures, for identifying treatments. Demonstrating the biological consistency between PDXCs and corresponding PDXs would validate the role of PDXCs as a useful model for rapid and comprehensive discovery of drug combinations targeting treatment-refractory cancers.

## Methods

### Generation of melanoma PDXs

#### Patient-derived tumour cells (PDCs)

Upon receiving human BRAF-metastatic melanoma tissue, samples were mechanically disrupted and cultured as described in Supplementary Methods.

#### Patient-derived xenograft generation and passaging

PDCs and PDXCs were passaged in vivo as described in Supplementary Methods. An example of a patient-derived culture used to seed a tumour is shown in Supplementary Fig. 1.

### High-throughput drug screening

#### Development of inhibitor screen

All PDXCs or PDCs were plated in 384-well low attachment round bottom microplates (Corning, Tewksbury, MA) within 3–7 days after processing the original tumour material or thawing frozen cells, and they were then allowed to acclimate for 3 days. The low attachment round bottom microplates, media conditions and acclimation period allowed for formation of tumourspheres before addition of drugs. Melanoma PDXCs were screened in an HTDS format (Supplementary Methods) against a 6-point concentration–response curve of drugs chosen primarily based on the following criteria: (1) The drug is FDA-approved or in clinical trials; (2) The drug is available for research purposes; (3) Pharmacokinetic (PK) data in humans are available. For most drugs, the 6-point concentration–response curve contained drug concentrations beginning with the highest concentration starting with the approximate maximum plasma concentration (*C*_max_) reported in published clinical trials (Supplementary Table 1). For a small percentage of drugs in historical screens, where there was significant HTDS data, the concentration was kept as long as it did not vary more than threefold of the published *C*_max_, the exceptions being vorinostat and gedatolisib which varied by 5–10-fold. The drug list used in this study and associated references are included (Supplementary Table 1).

#### Quantification of drug response

The resulting drug-response data were fitted using area under the pharmacological curve (AUC) and with the program Prism (GraphPad, San Diego, CA) using the formula ∆X*(Y1 + Y2)/2. Past studies and a recent large-scale screening effort demonstrate that AUC is more effective than either calculating the potency and efficacy of a drug across multiple cancer cell lines,^11,12^ since AUC takes into account both drug potency and efficacy in a single value. For ease of evaluation, we used a standard min–max normalisation equation^13^ to create a scoring scale similar to a typical grading scale. We transformed the AUC value to equal a value between 0 and 100: score = 100 × (1−(AUC−AUC_min_/AUC_max_−AUC_min_), where 0 is no effect and 100 represents killing of all the cells. Drugs that stimulate cell growth produce a score < 0. As part of our drug scoring platform, all the analyses are automated using specialised in-house VBA programmed Excel spreadsheets and Prism.

### Next-generation sequencing

Genomic DNA was extracted from fresh human and mouse PDX tissue samples as described in Supplementary Methods. A sequencing library targeting 212 amplicons in 48 genes was generated using the Illumina TruSeq Amplicon—Cancer Panel that provides pre-designed, optimised oligonucleotide probes for sequencing mutational hotspots in > 35 kilobases of target genomic sequence (Supplementary Methods).

### Treatment studies in vivo

Eight to ten-week-old female NSG mice (Jackson Laboratories, Sacramento, CA) bearing subcutaneous PDX tumours were randomised according to initial tumour volume. Mice where housed in a single ventilated cage in groups of four, provided environmental enrichment materials and free access to water and food. Mice were treated with either vehicle (control group), 20 mg/kg vemurafenib, 5 mg/kg cobimetinib or 5 mg/kg apitolisib, or specific combinations. All treatments were carried out in the home cage in a biosafety cabinet. Dosing ranges were based on previously published studies, and intraperitoneally (i.p.) dosing was chosen to standardised route of administration and vehicle formulation between all drugs. All drugs were diluted in the vehicle formulation: 4% DMSO, 4% Tween-80 and 92% saline. PDX tumour volumes were measured once per week via callipers, and treatment groups were blinded to measuring personnel. Treatment began when PDX tumour volumes reached ~100 mm^3^. PDX-bearing NSG mice were treated i.p. daily in the afternoon for 5 consecutive days for ~4 weeks for a total of 20 treatments or until PDX tumour volumes reached 2000 mm^3^. Tumours were measured approximately once weekly by an operator blinded to the treatment groups. Tumour volume was calculated as (length × width^2^)/2. The response was determined by comparing tumour volume change at time *t* to its baseline: % tumour volume change = ΔVol_*t*_ = 100% × ((V_*t*_−V_initial_)/V_initial_).^14^ The justification for the use two to four mice in the PDX and PDXC drug response comparison is explained in the Results section. Six to twelve mice were used for specific drug combination studies based on sample size calculations from initial studies. Animal number was increased in the drug combination groups to adjust for a smaller effect size expected when comparing single drug effects to drug combination effects, as opposed to comparisons made to the control group. At the completion of in vivo experiments, all animals were humanely killed using CO_2_ overdose followed by thoracotomy as outline by the American Veterinary Medical Association guidelines for the euthanasia of animals.

### Statistical analyses

Pearson correlations were determined, where appropriate. A Spearman's correlation coefficient (*p*) was calculated to assess the relationship between drug response of the PDXC and the corresponding PDX. PDX drug scores (for each cell line and drug pairing) were calculated as the average relative tumour growth (in percent) in the control relative to treatment. The average relative tumour growth was defined as the tumour volume at the final time divided by the initial tumour volume averaged over the total PDX realisations. The time span between initial and final tumour measurements was identical for the control and all treatment groups of a given PDX, and varied between 23 and 29 days. Differences in tumour growth between treatment groups were evaluated using two-way ANOVA repeated measures, and a Tukey’s multiple comparisons test. Statistical significance was defined as a *p*-value < 0.05.

## Results

### Establishment of PDX models from BRAF-mutant metastatic melanoma

Ten BRAF-mutant metastatic melanoma PDXs were established (Supplementary Table 2). For generation of PDXs, human tumour tissue samples were received within 1–2 h after resection. Samples were processed to create PDC and then implanted as cell suspensions (see the Methods section).

The ten samples were obtained from seven men and three women (Supplementary Table 2), with ages ranging from 49 to 79, and obtained from various sites, including lymph node, soft tissue and brain. Each patient had various treatment histories, including immunotherapy, BRAF inhibitors or BRAF + MEK inhibitors. As expected, PDXs had different growth rates even when the cell number injected for seeding of tumours was standardised (Supplementary Fig. 2). The metastatic melanoma samples collected, which were used to derive PDXs, reflect patient populations receiving the current standard of care, including immunotherapy and targeted therapy.

### Concordance of somatic mutations across patients and PDXs correlates with VAF

**Fig. 1:**
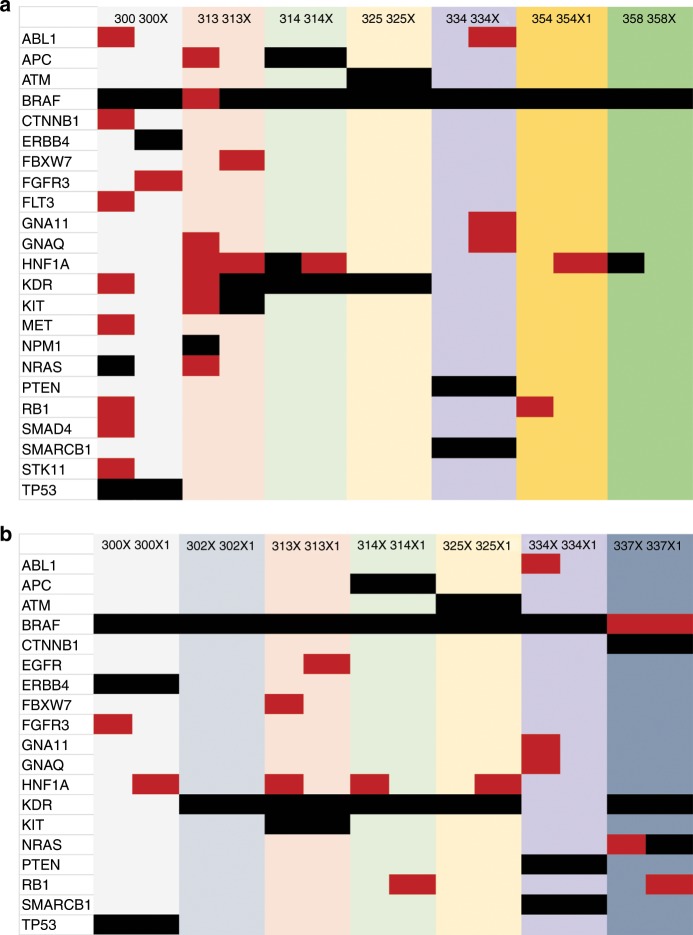
Comparison of SNV and insertion/deletion (ins/del) variants of DNA mutational hotspots from patient tumour and first- and second-generation PDX derivatives. **a** Patient tumour and corresponding first-generation (X) PDX, and **b** X and second-generation (X1) PDX generations were compared. The matched pairs are grouped using coloured columns, for example, MM300 with MM300X (light grey). The mutations identified are listed by row (far left column). Red indicates an allele frequency (AF) 5–24%. Black indicates an AF ≥ 25%.

Next-generation sequencing was performed to determine the DNA mutational profile across the original patient tumours and different generations of PDXs (Fig. 1; Supplementary Table 3). The nomenclature used for xenograft passaged tumours was X (first generation), X1 (second generation) and X2 (third generation), corresponding to serial passaging in vivo. A sequencing library targeting 212 amplicons in 48 genes was generated using the Illumina TruSeq Amplicon—Cancer Panel. BRAF mutations, either V600E or V600K, identified by standard clinical testing using paraffin-embedded patient tumour tissue (Supplementary Table 2), was confirmed in the fresh patient tumour and the corresponding first generation PDX (Fig. 1a; Supplementary Table 3), and identified in all subsequent generations of PDXs (Fig. 1b; Supplementary Table 3). In agreement with previously published melanoma Cancer Genome Atlas Network data,^2^ melanoma tumour tissues from the patients and their corresponding PDXs contained NRAS, PTEN, KIT, KDR and TP53 mutations. It should be noted that NF1, a common DNA mutation in melanoma,^2^ is not included in the standard Illumina panel and was therefore not evaluated.

The most striking finding was that an increase in VAF resulted in an increase in stability of specific mutations across the patient tumour, corresponding PDX (Fig. 1a; Supplementary Table 3), and subsequent generations of PDXs (Fig. 1b; Supplementary Table 3). Total concordance^15^ in patient tumour and first-generation PDX was 48% for VAF ≥ 10 and 88% for VAF ≥ 25% (Supplementary Table 3, Supplementary Methods). For first-generation PDXs versus second-generation PDXs, the total concordance was 91% for VAF ≥ 10 and 95% for VAF ≥ 25%. Our data demonstrate that PDXs harbour somatic mutations in cancer-driving genes observed in melanoma in accordance with TCGA data. Furthermore, the concordance of somatic mutations across patient tumour and matched PDXs, and additional PDX generations, increases as VAF increases. The fidelity of cancer-driving genes across human and PDX samples would suggest the conservation of responses to antitumor agents across derivatives.

### Evaluation of PDXC response to antitumor agents using HTDS

**Fig. 2:**
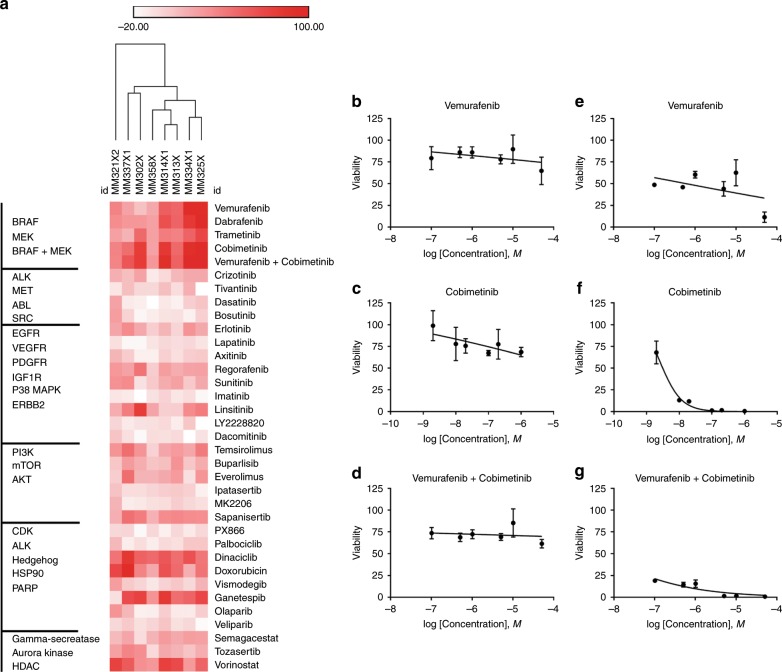
HTDS in eight BRAF-mutant melanoma PDXCs. **a** Hierarchical clustering of effects of single agent and vemurafenib + cobimetinib combination on melanoma cell viability/proliferation of eight PDXCs. Drug score of 0 equals no effect and 100 represents killing all the cells. Drugs that stimulate cell growth produce a score < 0. Darkest red of the heatmap indicates greatest inhibition of cell viability/proliferation and white equals no response. Drugs (right) were arranged based on the primary drug target (left). **b**–**d** Response of MM358X from a patient that progressed on BRAF and MEK inhibitors. **e**, **f** Response of MM314X from a patient not previously treated with BRAF and MEK inhibitors. Error bars are SD of *n* = 3 replicates.

Thirty-four antitumor agents were evaluated across eight metastatic melanoma PDXCs. These eight BRAF-mutant metastatic melanoma models were chosen for the remainder of the study, because they produced the most consistent tumour growth in mice and PDXC yield amendable to HTDS. Melanoma cells were cultured as three-dimensional tumourspheres in 384-well plates, treated with drugs for 3 days and assessed for cell viability (Fig. 2a; Supplementary Table 4). Since a precision medicine platform was being developed, we chose our maximum concentration in the assay to correspond to the maximum plasma concentration (*C*_max_) at the highest single dose recommended based on clinical trials when the information was available, and when the drug was not limited by solubility (see the Methods section). We developed a drug score based on calculating the area under the dose–response curve (AUC) with corresponding confidence intervals (CI). As cell viability/proliferation is decreased, the AUC is reduced.^11,12^ For ease of evaluation, we used a standard normalisation equation to create a scoring scale similar to a typical grading scale. We transformed the AUC value to equal a value between 0 and 100, where 0 is no effect and 100 represents killing of all the cells. Drugs that stimulate cell growth produce a score < 0.

As an example, responses to a BRAF inhibitor (vemurafenib), MEK inhibitor (cobimetinib) or the combination of both in MM358X are shown (Fig. 2b–d). MM358X is derived from a patient harbouring a BRAF-mutant melanoma that progressed on BRAF and MEK inhibitors. The PDXC showed resistance to inhibition by treatment with vemurafenib, cobimetinib and vemurafenib + cobimetinib. In addition, responses to vemurafenib, cobimetinib or the combination of both in MM314X are shown (Fig. 2e–g). MM314X is derived from a patient harbouring a BRAF-mutant melanoma not previously treated with BRAF and MEK inhibitors. The PDXC showed a marked response to vemurafenib, cobimetinib and vemurafenib + cobimetinib. Of the four patients that progressed on BRAF inhibitor therapy (MM321, MM337, MM302 and MM358), all PDXCs demonstrated resistance to vemurafenib in HTDS (Fig. 2a), when compared with the three previously untreated BRAF-mutant melanoma lines (MM313, MM314 and MM325) (Fig. 2a). The clinical history of MM334 is discussed in detail later as a case study. In addition to the previously described BRAF and MEK inhibitor responses, multiple tumours exhibited notable sensitivities to dinaciclib (CDK inhibitor), doxorubicin (DNA synthesis inhibitor), vorinostat (histone deacetylase inhibitor) and ganetespib (heat shock protein 90 inhibitor) (Fig. 2a). Overall, PDXCs recapitulate the resistance to BRAF and MEK inhibitor therapy observed in the patient, and PDXCs from treatment-naive BRAF-mutant melanoma patients showed a marked response to BRAF and MEK inhibitor therapy. The HTDS also identified notable sensitivities to additional antitumor agents that are FDA approved or in clinical trials, including dinaciclib, vorinostat, ganetespib and doxorubicin.

To determine whether drug response across derivatives was conserved, we evaluated drug sensitivities across various culture passages. We found a very high correlation between drug response using the same PDXC in different assays carried out within the same run, even when the plated cell number varied (Supplementary Fig. 3A, B). We also found a high correlation for drug sensitivities between different generations of PDXCs (Supplementary Fig. 3C–E) and between a PDC and corresponding PDXC (Supplementary Fig. 3F). We next aimed to determine whether the drug response between PDXCs and corresponding PDXs was conserved.

### Response of PDXCs to BRAF and MEK inhibition correlates with response observed in PDX models

**Fig. 3:**
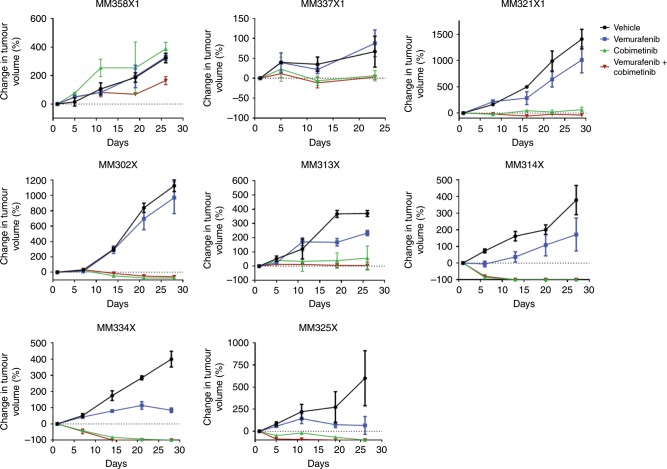
Treatment of multiple melanoma PDXs with vemurafenib, cobimetinib and vemurafenib + cobimetinib. Tumours from MM358X1, MM337X1, MM321X1, MM302X, MM313X, MM314X, MM334X and MM325X were generated in NSG mice (2–4 per group) by subcutaneous injection of 0.5 × 10^6^ cells. Animals with established tumours (average volume of 100 mm^3^) were randomised and treated with i.p. injections of vehicle, vemurafenib (20 mg/kg), cobimetinib (5 mg/kg) or the combination of vemurafenib (20 mg/kg) + cobimetinib (5 mg/kg) daily 5 days a week for an average of 3 weeks. Tumour volume (mm^3^) was calculated, and data were expressed as percent change in tumour volume. Error bars are SEM.

**Fig. 4:**
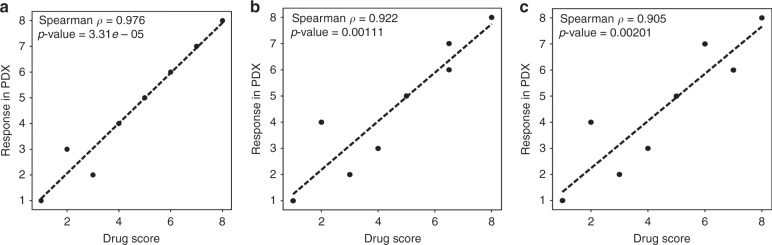
Correlation of drug response between patient-derived derivatives. Eight matched PDXCs and PDXs (MM358X1, MM337X1, MM321X1, MM302X, MM313X, MM314X, MM334X and MM325X) were treated with (**a**) vemurafenib, (**b**) cobimetinib or (**c**) vemurafenib + cobimetinib. A Spearman correlation analysis was performed comparing the response in PDXs to the drug score obtained in HTDS.

A few studies have suggested that highly robust targeted therapy drug responses observed in a PDXC can translate to some measure of response in a PDX.^9,10^ However, to date, no studies have demonstrated a statistical correlation between the magnitudes of targeted therapy drug response in HTDS utilising PDXC, when compared with the response in vivo in the corresponding PDX. We therefore investigated the correlation between the response to the BRAF inhibitor vemurafenib, the MEK inhibitor cobimetinib or the combination of both drugs, across eight PDXCs and corresponding PDX models (Fig. 3). Previously, a 1 × 1 × 1 model (one mouse, harbouring a single unique PDX, receiving one treatment condition) had been recommended to evaluate population responses of drug treatments across multiple PDXs.^14^ The responses can then be evaluated using modified RECIST criteria similar to that used in the clinic. This was expected to improve comparison of drug efficacy results between a PDX and the patient from which the PDX was derived. We found that implantation of a single tumour in a mouse for a single condition leads to unacceptable variation for analysis. Therefore, each group in our studies consisted of the average response from at least 2–4 mice. Modified RECIST criteria were used to evaluate the response.^14^ In all treatment groups, there was a significant correlation between the magnitude of drug response observed in the PDXC and corresponding PDX: vemurafenib (Fig. 4a), *p *= 0.98 (*p*-value = 0.03e−03); cobimetinib (Fig. 4b), *p* = 0.92 (*p*-value = 1.11e−03); and vemurafenib + cobimetinib (Fig. 4c), *p* = 0.91 (*p*-value = 2.01e−03). Therefore, using BRAF and MEK inhibitors, both individually and in combination, we found a high correlation between the drug response in PDXCs and corresponding PDXs.

### Patterns of response to BRAF and MEK inhibitors following in vivo development of drug resistance

**Fig. 5:**
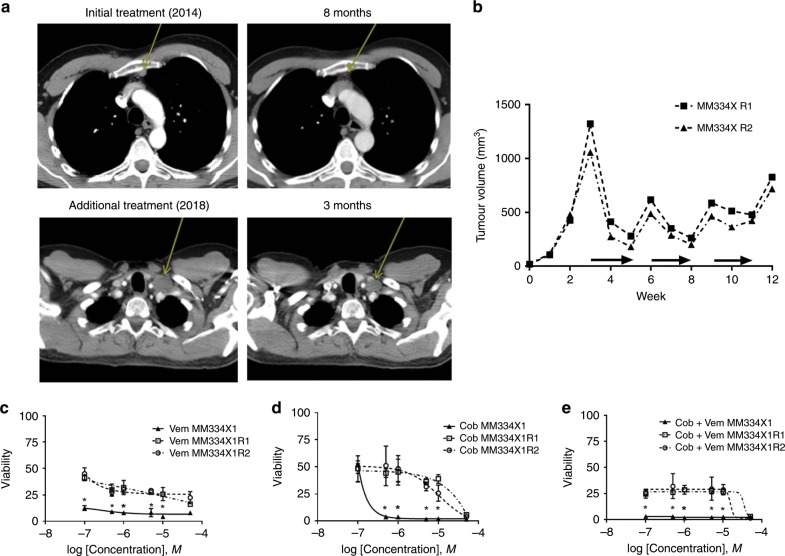
Biological consistency of producing targeted therapy responses in PDXs. **a** Upper left, CT scan of patient MM334 presenting with a metastatic melanoma to the right inguinal lymph node (yellow arrow). Upper right, remission of tumour after treatment with BRAF and MEK inhibitors (yellow arrow). Lower left, recurrence of the melanoma in the left subclavicular lymph node. Lower right, tumour persisting after BRAF and MEK inhibitor treatment. **b** Resistance training of two MM334 PDX tumours. Horizontal arrows indicate periods of treatment with BRAF and MEK inhibitors. HTDS response to (**c**) vemurafenib, (**d**) cobimetinib and (**e**) cobimetinib + vemurafenib in pre- (solid line) and post-resistance (dashed lines) MM334 PDXC. (*) indicates statistically significant differences between resistant and control groups (*p* < 0.05).

We next explored the biological consistency of response to targeted therapy between PDX and corresponding PDXC models in an in vivo model of drug resistance. We selected melanoma MM334X, a PDX that originally demonstrated a significant response to BRAF and MEK therapy in vivo. Patient MM334 was originally treated with BRAF + MEK therapy and had a dramatic and durable response after multiple cycles of therapy (Fig. 5a upper panels; Supplementary Fig. 4), resulting in treatment discontinuation. Seven months later, the patient progressed, with metastatic disease also involving the brain. The brain metastasis was resected and processed for PDX generation. Over the course of the next 3 years, the patient was treated with multiple rounds of stereotactic radiosurgery and a short course of immunotherapy. The detailed patient history timeline is presented in Supplementary Fig. 4. When the tumour recurred again in the brain and progression was not halted with radiation or immunotherapy, the patient was again treated with BRAF + MEK inhibitors, and a response to therapy was observed (Fig. 5a lower panels).

We subsequently created a PDX model of targeted therapy resistance using MM334 in multiple individual mice by sequential on/off dosing with vemurafenib + cobimetinib (Fig. 5b). We aimed to assess the biological consistency in responses observed to targeted drugs across different mice harbouring the same initial tumour. Initially, PDXs derived from MM334X showed a robust response to BRAF and MEK therapy (Fig. 5b). After three treatment rounds, all tumours became resistant to the combination of vemurafenib + cobimetinib (Fig. 5b). We removed the PDX from two individual mice (R1 and R2) and performed HTDS on each of the corresponding PDXCs. HTDS revealed reductions in potency and efficacy for the single agents and drug combination in comparison with the original PDX (Fig. 5c–e). Based on the pharmacological screen, the PDXC derived from each individual tumour showed an identical reduction in response to drug treatment. These data demonstrate that prolonged treatment of individual mice bearing the same PDX with BRAF and MEK inhibitor therapy leads to an identical reduction in drug response. In addition, the reduction in drug response was consistently observed in the corresponding culture models (PDXC).

### HTDS of BRAF inhibitor-resistant melanoma identifies novel targetable drug combinations

**Fig. 6:**
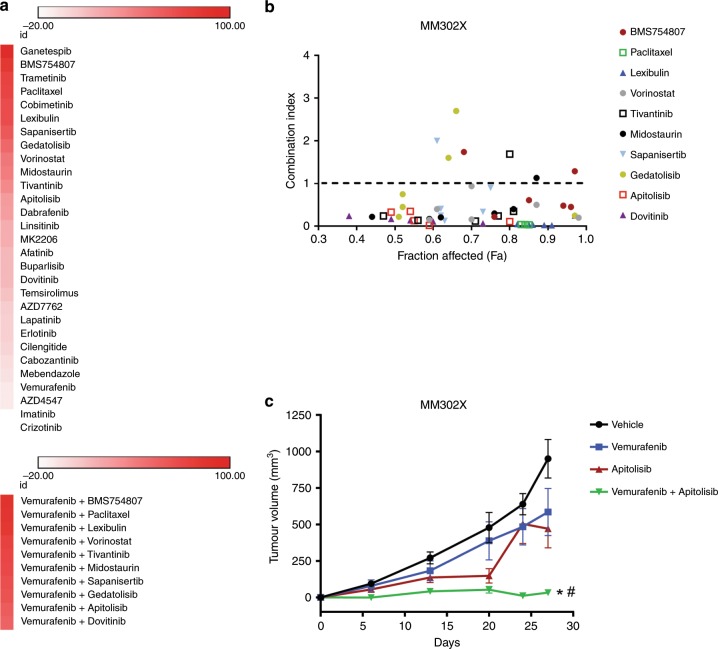
Identification of multiple drug combinations that inhibit the growth of BRAF inhibitor-resistant melanoma. **a** Response of single agent or drug combinations on melanoma cell viability/proliferation in MM302X PDXCs. Drug score of 0 equals no effect and 100 represents killing all the cells. Drugs that stimulate cell growth produce a score < 0. Darkest red of the heatmap indicates greatest inhibition of cell viability/proliferation, and white equals no response. **b** CI values (range 0–4) for drug combinations in MM302X PDXCs. A CI value of < 1, 1 and > 1 indicates synergism, additivity and antagonism, respectively. **c** Tumours from MM302X were generated in NSG mice (6–12 per group) by subcutaneous injection of 0.5 × 10^6^ cells. Animals with established tumours (average volume of 100 mm^3^) were randomised and treated with i.p. injections of vehicle, vemurafenib (20 mg/kg), apitolisib (5 mg/kg) or the combination of vemurafenib (20 mg/kg) + apitolisib (5 mg/kg) daily 5 days a week for 4 weeks. Tumour volume (mm^3^) was calculated, and data was expressed as % change in tumour volume. Error bars are SEM. * and ^#^ indicates statistically significant differences between vehicle and vemurafenib + apitolisib, or apitolisib and vemurafenib + apitolisib, respectively (*p* < 0.05).

The fidelity of cancer-driving mutations, pharmacological response and development of resistance observed between PDXCs and PDXs suggests that PDXCs alone may constitute a suitable precision medicine platform for discovering novel drug combinations to target BRAF inhibitor- resistant metastatic melanoma. Since PDXCs are an expandable resource, we ran an extensive HTDS on the BRAF inhibitor- and immune therapy-resistant PDXC MM302X in order to discover novel combinations of drugs to overcome BRAF-inhibitor resistance. MM302 was derived from a patient that was initially placed on BRAF + MEK-targeted therapy, but had an adverse reaction to the MEK inhibitor; therefore, the patient continued treatment on BRAF inhibitor alone until the tumour progressed. The patient was also treated with immunotherapy and also progressed. MM302X cells were treated with a full concentration response curve of 29 drugs, or the full concentration response curve of the drugs combined with a single concentration of vemurafenib (5 μM, 10% *C*_max_) (Fig. 6a). Responses in the presence or absence of vemurafenib were compared statistically using AUC and corresponding confidence limits (see the Methods section). The top ten drug combinations that performed better than single agents included two agents targeting microtubules (paclitaxel and lexibulin), three drugs that targeted mTOR or PI3K/mTOR (everolimus, apitolisib and sapanisertib), and drugs inhibiting IGF1R (BMS754807), HDAC (vorinostat), MET (tivantinib), RTK (dovitinib) and PKC (midostaurin) (Fig. 6a; Supplementary Table 5). We further analysed the compounds for drug interactions using the combination index (CI) (Fig. 6b). All the inhibitors demonstrated multiple synergistic interactions (below dash line) across multiple concentration ranges, with some additive (CI = 1) and antagonistic (above dash line) interactions observed; the latter is expected when testing a range of concentrations when combining two drugs.^16^ The top ten drug combinations were tested again in two additional BRAF inhibitor-resistant PDXCs, MM358X (Supplementary Fig. 5A, C; Supplementary Table 5) and MM337X (Supplementary Fig. 5B, D; Supplementary Table 5). With the exception of midostaurin, in MM358X the drug combinations were also more active than the drugs alone. However, the majority of synergistic interactions were observed only when inhibition of cell viability/proliferation exceeded 50% (fraction affected) (Supplementary Fig. 5C). In comparison to MM302X and MM358X, MM337X was more resistant to single drug activity and drug combinations (Supplementary Fig. 5B, D; Supplementary Table 5).

Multiple drugs targeting the mTOR or PI3K/mTOR pathway show synergetic interactions when combined with vemurafenib. As a result, we chose the combination of vemurafenib + apitolisib to validate drug response in vivo (Fig. 6c). Apitolisib is a dual PI3K/mTOR inhibitor.^17,18^ Vemurafenib + apitolisib demonstrated low CI scores across multiple concentrations in MM302X. Mice-bearing tumours derived from MM302X were treated with vehicle, vemurafenib, apitolisib or vemurafenib + apitolisib. As expected, the MM302X-derived tumours were resistant to vemurafenib alone. While apitolisib alone produced some initial antitumor activity, the tumour became resistant over time. The combination of vemurafenib + apitolisib produced a robust and sustained inhibition of tumour growth. These data demonstrate the utility of using the HTDS platform and PDXCs to discover multiple novel drug combinations to overcome BRAF-inhibitor resistance in BRAF-mutant melanoma.

## Discussion

In the development of effective personalised therapy platforms for cancer patients, PDX models have become the preferred preclinical model both in academic research and in industry.^4,6,19–22^ A major drawback of PDX technology is the time and cost to test the inhibitory activity of multiple single-drug therapies, and combination therapies. It is therefore important to determine whether the response to specific targeted therapies in derivatives of PDXs, such as PDXCs, show a direct correlation to the response observed in vivo. If they do, PDXCs used in HTDS format represent a high speed and lower cost opportunity to study single agent and drug combination therapy.

Past studies have shown that a robust single agent or combination therapy drug response in culture (PDXCs) led to some measure of inhibitory activity in vivo in the corresponding PDXs.^9,20^ However, we are not aware of any published studies to date that determined whether there is a direct statistical correlation in the magnitude of drug responses, at all response levels, when single and combination therapies are compared across multiple PDXCs and corresponding PDXs.

To answer this question, we first developed a PDX library of BRAF-mutant metastatic melanoma and observed a correlation between VAF and increased stability of driver mutations across the patient tumour, corresponding PDX and subsequent generations of PDXs for multiple genes (e.g., BRAF, KDR, TP53 and PTEN). A recent pan-cancer analysis has shown that high somatic VAF in tumours is associated with cancer-driving genes.^22^

Using HTDS, we next evaluated the response of eight metastatic melanoma PDXCs to 34 antitumor agents that are either FDA approved or in clinical trials. As part of our HTDS platform, we incorporated the use of three-dimensional tumourspheres (PDXC) and clinically relevant drug exposures. The tumourspheres better approximate drug penetrance levels in a three-dimensional tumour. Setting the highest drug concentration used in HTDS to the approximate *C*_max_ reported in published clinical trials was expected to improve clinical translation and reduce false positive rates.^23^ The combination of using tumourspheres and relevant drug exposures was also expected to translate to improved correlation between responses in culture and in vivo. In addition to evaluation of response to BRAF and MEK inhibitors, HTDS identified notable sensitivities to single agent dinaciclib, vorinostat, ganetespib and doxorubicin across multiple BRAF-mutant metastatic melanoma PDXCs. Dinaciclib is an inhibitor of CDK1/2/5/9, and has demonstrated clinical activity in multiple myeloma.^24^ It has been shown to produce tumour regression in human melanoma xenografts.^25^ Vorinostat is a histone deacetylase (HDAC) inhibitor that has demonstrated antitumor activity in patients with cancer.^26^ HDAC therapy alone or in combination with BRAF and/or MEK inhibitors has been suggested as a treatment for BRAF-mutant melanoma through multiple different mechanisms.^27–29^ Ganetespib is a heat shock protein 90 (HSP90) inhibitor^30^ and has demonstrated antitumor activity in a variety of cancer types, and is currently being evaluated in multiple clinical trials.^31^ Ganetespib has been shown to inhibit survival of melanoma with acquired resistance to BRAF inhibition through multiple pathways, including inhibition of extracellular signal-regulated kinase (ERK) signalling.^32,33^ Doxorubicin is a classical chemotherapy agent that works in part through interfering with DNA synthesis, and used for a variety of cancers, though not commonly used to treat melanoma. A phase II clinical trial in 30 melanoma patients utilised a pegylated liposomal form of the drug to decrease toxicity and identified a subpopulation of responders without a significant improvement in survival.^34^ A goal of the PDXCs, PDXs and other precision medicine preclinical models is to provide a preclinical assessment of patient response to specific drugs and refine clinical trials by identifying relevant “responder” patient populations as well as to develop biomarkers of therapeutic response^35^ and discover mechanisms of resistance.^36^

In the HTDS, PDXCs derived from patients harbouring a BRAF-mutant melanoma that progressed on BRAF and MEK inhibitors showed resistance to these treatments, whereas PDXCs derived from treatment-naive patients showed a marked response to these treatments. Similar results were found in vivo. This demonstrates a significant correlation between drug response in PDXCs, PDXs and patients. We also compared drug response in eight metastatic melanoma PDXCs and corresponding PDXs treated with vemurafenib, cobimetinib and vemurafenib + cobimetinib, and found a strong correlation between all levels of drug responses measured between PDXC and the corresponding PDX. The biological consistency between PDXCs and PDXs was also evaluated in the context of in vivo development of resistance to BRAF + MEK inhibitor therapy. Tumours derived from the same initial PDX were treated with vemurafenib + cobimetinib for multiple cycles until the tumour progressed on therapy. Based on the pharmacological screen, each individual tumour showed identical resistance to the treatments, demonstrating the biological consistency of the models.

A recent investigation provided evidence that particular copy-number alterations (CNAs) acquired during PDX passaging differed from those acquired during tumour evolution in patients, and that CNAs for cancer cell lines and PDXs were similar.^37^ Furthermore, the data suggested that model-acquired CNAs predominantly result from clonal dynamics, rather than from genomic instability. The authors cautioned that genetic drift is an inevitable part of cancer progression and can differ depending on the specific microenvironment, namely the patient microenvironment versus the mouse. While all preclinical models have specific weaknesses, numerous studies utilising PDX models,^6–8^ including the Izumchenko et al.^4^ study, show a high correlation rate between PDXs and patient response; therefore, expansion in the mouse microenvironment still results in tumours with significant utility for precision medicine platforms.

Given the high correlation between responses observed between PDXs and patients,^4^ and high correlation of drug response between PDXCs and PDXs, for specific drugs PDXCs may prove useful to predict responses in patients. Pharmacogenomic evaluation of PDXC has also shown that these derivatives recapitulate known mechanisms of both drug sensitivity and resistance.^10^ A recent investigation demonstrated that drug response in patient-derived tumour cells (PDCs) predict the response of the patient’s tumour retrospectively for specific drugs.^38^ While this investigation did not evaluate responses in melanoma, it does demonstrate the feasibility of making treatment predictions using PDCs. In this study^38^ and in a large-scale analysis of PDC derived from haematological malignancies,^39^ investigators demonstrated that pharmacogenomic analysis allowed for the identification of novel treatments and markers of drug sensitivity. The major drawback with PDC is the initial tumour is often too small to create enough viable PDCs for screening, and extensive drug combination studies are generally not feasible. Importantly, without creating a renewal resource, namely PDXs and PDXCs, one cannot guarantee opportunity for future investigations.

As a result of the biologically consistency between PDXCs and corresponding PDXs, we used the HTDS platform and PDXCs to discover multiple novel combinations of drugs to overcome BRAF-inhibitor resistance. Across three BRAF inhibitor-resistant PDXCs, pathways identified as therapeutic targets when combined with BRAF inhibition included PI3K/mTOR, microtubules, HDAC, MET and RTK. Notably, the IGF1R inhibitor BMS754807 in combination with BRAF inhibition produced a robust inhibitory response in two of the three BRAF-resistant PDXCs. We validated responses observed in a PDXC in a corresponding PDX by evaluating apitolisib in combination with vemurafenib. As predicted by HTDS, the combination of vemurafenib and apitolisib produced a robust and sustained inhibition of tumour growth in vivo. In agreement with our findings with BRAF inhibitor-resistant melanoma, it has been shown using BRAF/MEK-resistant melanoma PDX and next-generation sequencing that the MAPK pathway activation represents a common mechanism of resistance to BRAF/MEK therapy.^40^ Inhibition of MET, RTK and IGF1R can each impact MAPK signalling at various levels, although the upstream inhibition of MAPK through IGF1R appears to be more efficient, at least in two of the three BRAF inhibitor-resistant PDXCs. Finally, in BRAF inhibitor-sensitive cell lines, targeting HDAC has been shown to enhance efficacy of BRAF inhibitors through production of DNA damage.^27^ Our data, using a PDX/PDXC platform, suggest the potential utility of this drug combination in the resistance setting.

In summary, utilising a HTDS platform, we found a direct correlation between magnitude of drug response in short-term PDXCs and corresponding PDX models for BRAF, MEK and BRAF + MEK inhibitor. We also identify multiple novel drug combinations that may overcome BRAF inhibitor-resistance in melanoma. Melanoma has a high mutational burden, and a significant proportion of melanomas adapt to current therapies and progress, at which point (combinatorial) targeted therapies are likely a next option. Our findings also underscore the potential usefulness of this platform to discover additional novel combination therapies in other treatment-resistant cancer  populations/cohorts. Our results provide a framework for identifying and validating drugs that have a high correlation of response between PDXCs and PDXs. In addition, biological consistency is observed when developing models of BRAF + MEK inhibitor-resistance from PDXs. Systematically identifying antitumor agent responses that correlate between PDXCs and PDXs could support precision medicine approaches for cancer patients by providing a high-speed and low-cost opportunity to study the activity of multiple single agents and drug combinations. The latter is particularly important because aggressive tumours often do not respond to single-agent therapy.

## Supplementary information

Supplementary Material

## Data Availability

The data that support the findings of this study are available from the corresponding author upon reasonable request.
