# Supplementary material for: Drug responses are conserved across patient-derived xenograft models of melanoma leading to identification of novel drug combination therapies
Source: Br J Cancer. 2019 Dec 20;122(5):648–57. doi: 10.1038/s41416-019-0696-y (PMC7054294; doi:10.1038/s41416-019-0696-y)
Supplement: Supplementary file 1 — Supplementary Material [file 41416_2019_696_MOESM1_ESM.pdf]

**SUPPLEMENTARY METHODS**

*Patient-derived tumor cells (PDC)*

Upon receiving human BRAF-metastatic melanoma tissue, samples were mechanically disrupted and incubated in DMEM/F12 media (Gibco, Gaithersburg, MD) supplemented with B27 serum-free (Gibco, Gaithersburg, MD) and penicillin/streptomycin 10,000 u/mL (Gibco, Gaithersburg, MD) containing 0.25%, trypsin/EDTA (Gibco, Gaithersburg, MD), collagenase/hyaluronidase (StemCell Tech, Vancouver, BC), 1 μg/mL deoxyribonuclease I (BioChem, King of Prussia, PA) for 30 min and passed through a 40 μM cell strainer. Cell pellets were washed with phosphate buffered saline (PBS) without calcium and magnesium (Gibco, Gaithersburg, MD) and centrifuged at 300 x g for 5 min. Cell pellets were inspected for erythrocyte contamination and if present lysed utilizing lysis solution as per manufacturer's protocol (Miltenyi Biotech, Sunnyvale, CA). PDCs were cultured in a T75 ultra low attachment flasks (Corning, Tewksbury, MA) with DMEM/F12 media supplemented with B27, penicillin/streptomycin (10,000 u/mL), 50 μg/mL human epidermal growth factor (hEGF) (Peprotech, Rocky Hill, NJ), 50 μg/mL human fibroblast growth factor (hFGF) (Peprotech, Rocky Hill, NJ), and 5μg/mL heparin (Sigma, Carlsbad, CA). This media support the growth of tumorspheres (Supplementary Figure 1) but not stromal components. PDC were cultured for 3-7 days and then used to generate PDX or cryopreserved utilizing CellBanker2 without serum (AmsBio, Cambridge, MA).

*Patient-derived xenograft generation and passaging*

1x$10^6$ PDCs in 1:1 PBS:Matrigel (Corning, Tewksbury, MA) were injected subcutaneously (SQ) into the flank of female NOD *SCID* gamma mice (NSG) (Jackson Laboratories, Sacramento, CA). Before reaching 2000 $mm^3$ in volume, tumors were harvested and PDXC were processed in a manner identical to PDCs. PDXC were then either re-injected subcutaneously into the flank of NSG mice for an *in vivo* experiment, further *in vivo* passaging, plated for HTDS, or cryopreserved utilizing CellBanker2 without serum (AmsBio, Cambridge, MA). All PDXC were authenticated using short tandem repeat analysis (ATCC) and were mycoplasma-free. *In vivo* studies were carried out in accordance with the National Institutes of Health guidelines, Health Research Extension Act of 1985 and the Public Health Service Policy on Humane Care and Use of Laboratory Animals (Policy), Office of Laboratory Animal Welfare assurance, and an approved Institutional Animal Care and Use Committee (IACUC) protocol.

*HTDS screening platform*

An Excel-based .csv for dosing PDXCs was automatically generated using specialized in-house VBA programmed Excel spreadsheets and submitted to the company Transcriptic (Menlo Park, CA). The protocol was then translated into a python script to generate autoprotocol, a data structure used to instruct a workcell to execute experiments. Compatible plates and tubes were supplied to the workcell and a robotic arm then moves the containers to and from the appropriate instruments without human intervention. Drugs were supplied to Transcriptic at 1000X their effective concentrations in standard microcentrifuge tubes. These were transferred to a 384-acoustic liquid handler source plate that was then moved to a multi-channel liquid handler to serially dilute the drugs. The standard dilution protocol was 1X, 0.2X, 0.1X, 0.02X, 0.01X, 0.002X, where 1X is equal to the $C_{max}$ of the drug (Supplementary Table 1).

This source plate was used to dose 384-well microplates containing PDXC samples. Before dosing, the general health of the cells in a control lane are visually assessed. Using a simple .csv well map, the acoustic liquid handler automatically transferred each drug at each concentration from the source plate to the appropriate location on each of the cell-containing 384-well microplates. The drugged cell lines were then placed in an incubator for 72 hours, after which the CellTiter-Glo (Promega, San Luis Obispo, CA) assay was used to quantify cell viability/proliferation. Data from luminescence reads were subsequently rendered graphically in Transcriptic's web interface and made available for download in .csv format for further data analysis.

*Culturing conditions for PDXC in HTDS*

Blood serum albumin (BSA) in fetal bovine serum (FBS) is primarily responsible for binding and reducing activity of many drugs (11). Unlike standard cell lines, media used to propagate PDXCs does not contain FBS in order to more readily preserve the genomic and biological characteristics of the original tumor (12,13). Therefore, 0.1% purified BSA was added to PDC and PDXC growth media for HTDS in order that results could be more readily cross-compared to past and future studies using standard cell culturing techniques (14). Higher concentrations of BSA were not used because they significantly increase the cleaning maintenance of the automated cell dispenser cassettes.

*Drug storage and preparation*

Drugs were obtained from Selleckchem (Houston, TX) and master stocks were primarily made at half maximum solubility with DMSO or water diluent per manufacturer specifications for molecular weight, diluent and solubility. Drug stocks were then diluted to clinically relevant

maximum concentrations and transferred to 384-well stock drug plates at 65 μL. To prevent multiple freeze-thaw cycles of the master stocks, drugs are first aliquoted to a master plate, and then multiple working daughter 384-well plates were made, which were discarded monthly. For drug dosing, drugs from working passage plates were transferred at 25 nL to individual wells containing a 25 μL cell suspension media pre-seeded with tumorspheres. All drugs and drug plates were stored at -80 ℃. All drugs and drug plates were set to have one-year shelf life and discarded upon passing this point.

*Next Generation Sequencing*

Isolation of genomic DNA from fresh human and mouse PDX tissue samples was performed using DNeasy tissue kit (Qiagen, Redwood City, CA) after tissue disruption using ruptor disposable probes, and DNA was quantified using PicoGreen (Thermo Fisher Scientific, South San Francisco, CA). The integrity was determined using agarose gels.

Illumina (Foster City, CA) MiSeq 2x151 base paired-end sequencing results were validated to detect single-nucleotide variant (SNV) and insertion/deletion (indel) variants at 5% allelic frequency or higher in target regions with sufficient read coverage. The gene targets covered by this assay were as follows: ABL1, AKT1, ALK, APC, ATM, BRAF, CDH1, CDKN2A, CSF1R, CTNNB1, EGFR, ERBB2, ERBB4, FBXW7, FGFR1, FGFR2, FGFR3, FLT3, GNA11, GNAQ, GNAS, HNF1A, HRAS, IDH1, JAK2, JAK3, KDR, KIT, KRAS, MET, MLH1, MPL, NOTCH1, NPM1, NRAS, PDGFRA, PIK3CA, PTEN, PTPN11, RB1, RET, SMAD4, SMARCB1, SMO, SRC, STK11, TP53, VHL. Concordance between the original sample and its derivative was calculated as described by Calapre et al (17):

Concordance: C = 100% * $(x/ y)$

$x$ = number of variants confirmed in both the initial tumor tissue AND the derivative tissue

$y$ = number of similar variants confirmed in initial tumor tissue OR the derivative tissue

When there is a threshold (0, 10%, 25%) then there are different definitions of when variants are confirmed in both the initial tumor tissue and the derivative mouse tissue. The variants are confirmed in both tissues if the variant is above the threshold in at least one tissue and above zero in the other tissue.

**SUPPLEMENTARY FIGURES**

**Supplementary Figure 1. Tumorspheres.** Metastatic melanoma MM337X1 were cultured in T75 ultra low attachment flasks with DMEM/F12 media supplemented with B27, penicillin/streptomycin, hEGF, hFGF, and heparin. Microphotographs were captured 72 hours following initial cell plating utilizing a Zeiss AxioObserver Z1 with a 10X objective. Bar, 300 μm.

**Supplementary Figure 2. PDX tumor growth rate.** PDXCs were injected *in vivo* into the flank of NSG mice and monitored for growth. The number of days that were required to reach 500 $mm^3$ is indicated, illustrating the variability in growth rates between patient samples.

**Supplementary Figure 3. Correlation of HTDS response between PDXC generations.** The ability of multiple drugs to inhibit cell viability/proliferation was assessed. Scatter plots of the

correlation in drug score between different PDXCs, and PDCs and corresponding PDXCs. **A)** 500 versus 1000 MM300X cells, **B)** 250 versus 1000 MM300X cells, **C)** MM302X cells screened on 10/30 versus 11/6, **D)** MM337X cells screened on 1/21 versus 2/8, **E)** MM302X cells versus MM302X3 cells, and **F)** MM358 (PDC) versus MM358X (PDXC). A Pearson's correlation coefficiency test was performed.

**Supplementary Figure 4. Patient treatment history timeline for MM334.** Treatment with BRAF (dabrafenib) and MEK (trametinib) inhibitors is highlighted (red). Responses to treatments are also highlighted (* blue). MM334 was collected on October 2015 (red arrow): complete response (CR) and stable disease (SD) are indicated.

**Supplementary Figure 5. Drug combinations that inhibit the growth of BRAF inhibitor-resistant melanoma.** Response of single agent or drug combinations on melanoma cell viability/proliferation in **A)** MM358X PDXC and **B)** MM337X PDXC. Drug score of 0 equals no effect and 100 represents killing all the cells. Drugs that stimulate cell growth produce a score < 0. Darkest red of the heat map indicates greatest inhibition of cell viability/proliferation and white equals no response. **C)** CI values (range 0-4) for drug combinations in MM358X PDXC and **D)** MM337X PDXC . A CI value of <1, 1 and >1 indicates, synergism, additivity and antagonism respectively.

## SUPPLEMENTARY TABLES

**Supplementary Table 1. Drug list.** Drugs list with corresponding $C_{max}$ values and PubMed references. $C_{max}$: maximum plasma concentration; PMID: PubMed identifier; PMCID: PubMed identifier for all works published in the free-to-access PubMed Central.

**Supplementary Table 2. Clinical annotation and genetic information for patients.** M: male, F: Female. BRAF status was identified using standard clinical analysis using a CLIA certified test. GM-CSF: Granulocyte-macrophage colony-stimulating factor. All samples were collect after the specified therapy.

**Supplementary Table 3. Comparison of single-nucleotide variant (SNV) and insertion/deletion (ins/del) variants of DNA mutational hotspots from patient tumors and first (X) and second (X1) generation PDX derivatives.** Patient tumors and corresponding first generation (X) PDXs as well as X and X1 PDXs where compared. The match pairs are grouped using colored columns. The mutations identified are listed by row. Allele frequencies are also listed by row (for example 0.14 = 14%). Allele frequencies of SNV or ins/del that are present in matched samples are highlighted in yellow. Concordance (%) = ($x \div y \times 100$). Concordance at ≥ 10% AF and ≥ 25% AF was calculated for each matched pair and for all samples (Total Concordance).

**Supplementary Table 4. Drug score and AUC for HTDS in 8 BRAF mutant melanoma PDXCs.** Drug scores and AUC values were calculated for single agents and vemurafenib + combimetinib

when assessing melanoma cell viability/proliferation. Drug score of 0 equals no effect and 100 represents killing all the cells. Drugs that stimulate cell growth produce a score < 0. An AUC value of 0 represents killing all the cells, a value of 270 represents no cell kill, values > 270 represent stimulation of cell growth.

**Supplementary Table 5. Drug score and AUC for single agents and drug combinations in PDXC.** Drug scores and AUC values were calculated for PDXC MM302X, MM358X, and MM337X. Drug score of 0 equals no effect and 100 represents killing all the cells. Drugs that stimulate cell growth produce a score < 0. An AUC value of 0 represents killing all the cells, a value of 270 represents no cell kill, values > 270 represent stimulation of cell growth. Drug responses in the presence or absence of vemurafenib were compared statistically using AUC and corresponding confidence limits.

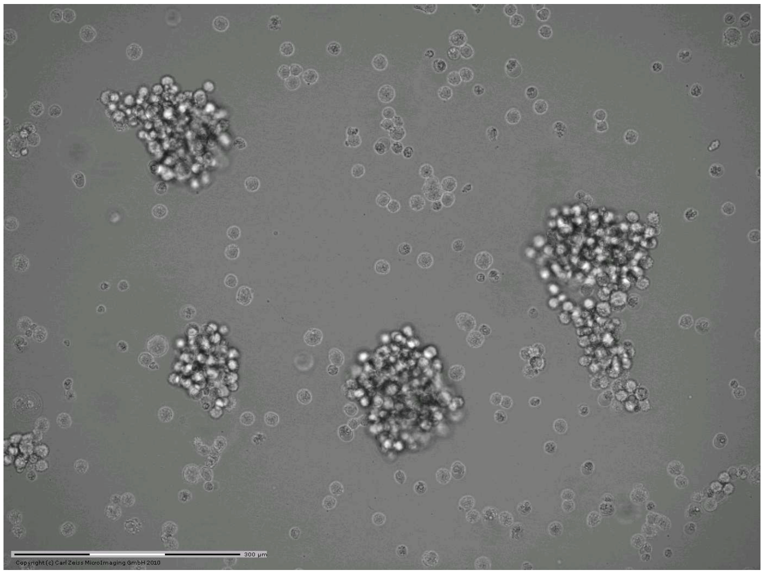


Supplementary Figure 1

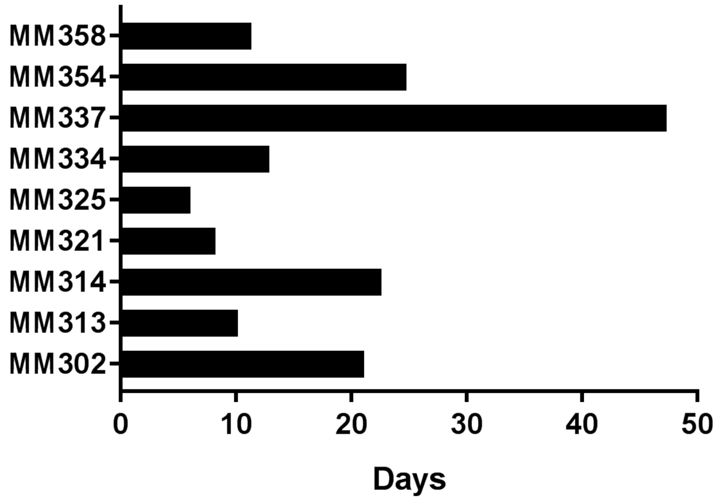


Supplementary Figure 2

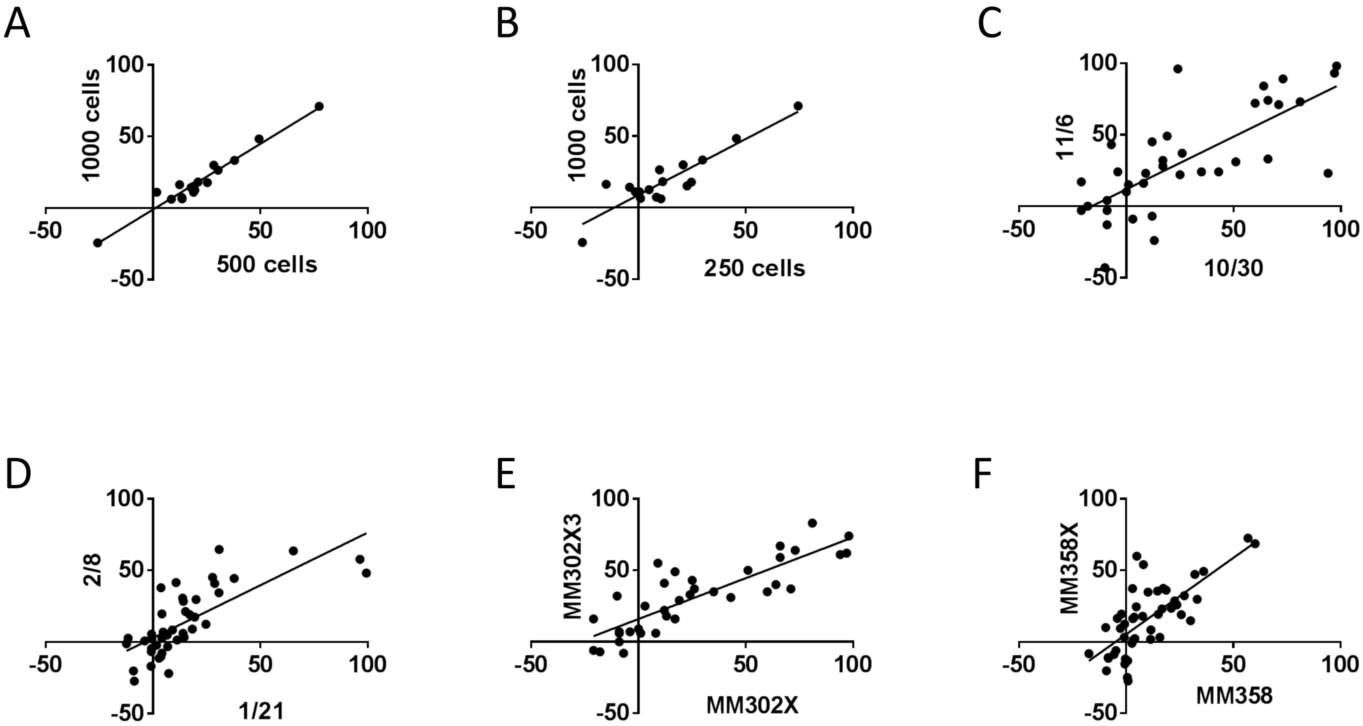


Supplementary Figure 3

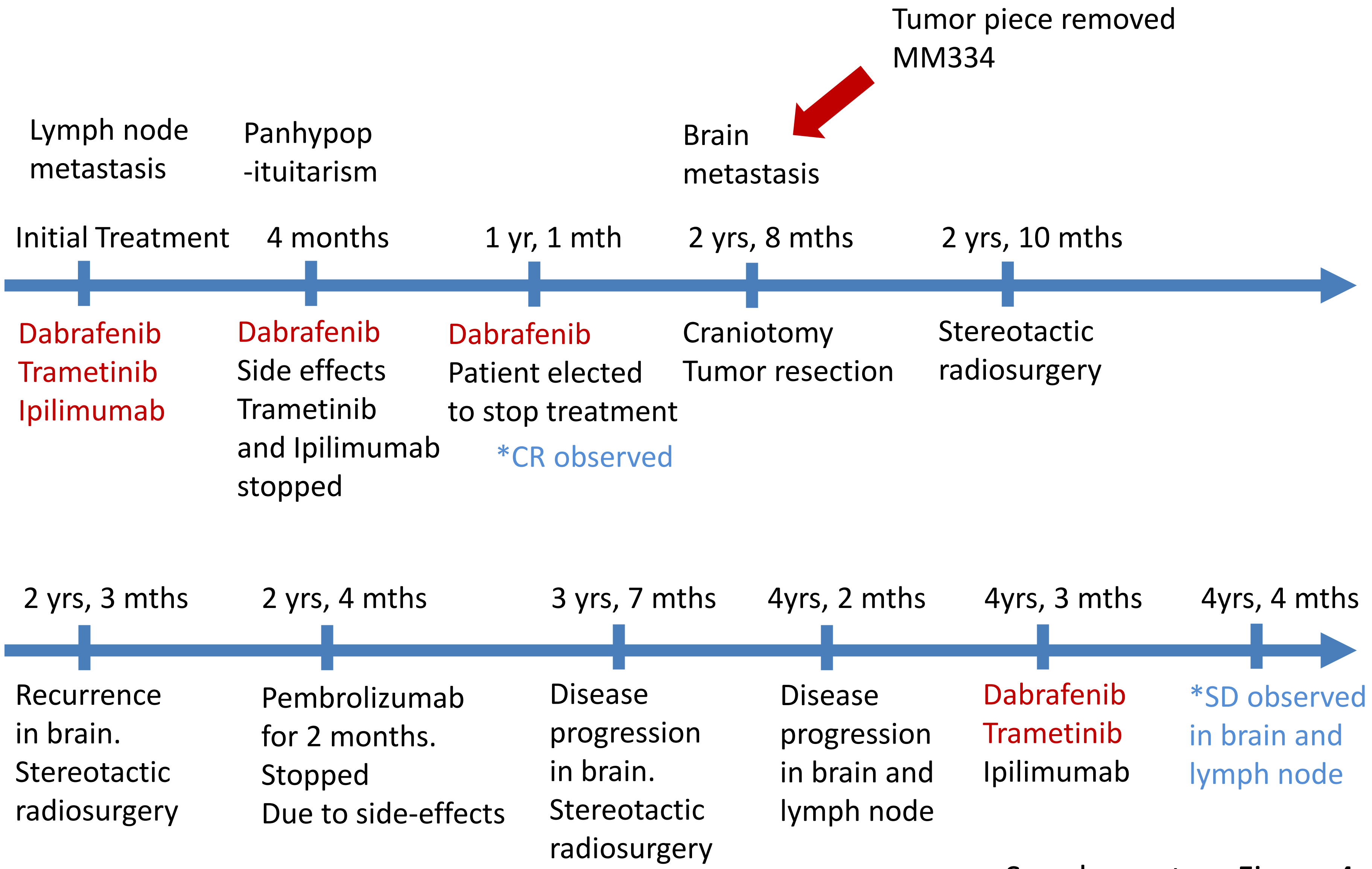


Supplementary Figure 4

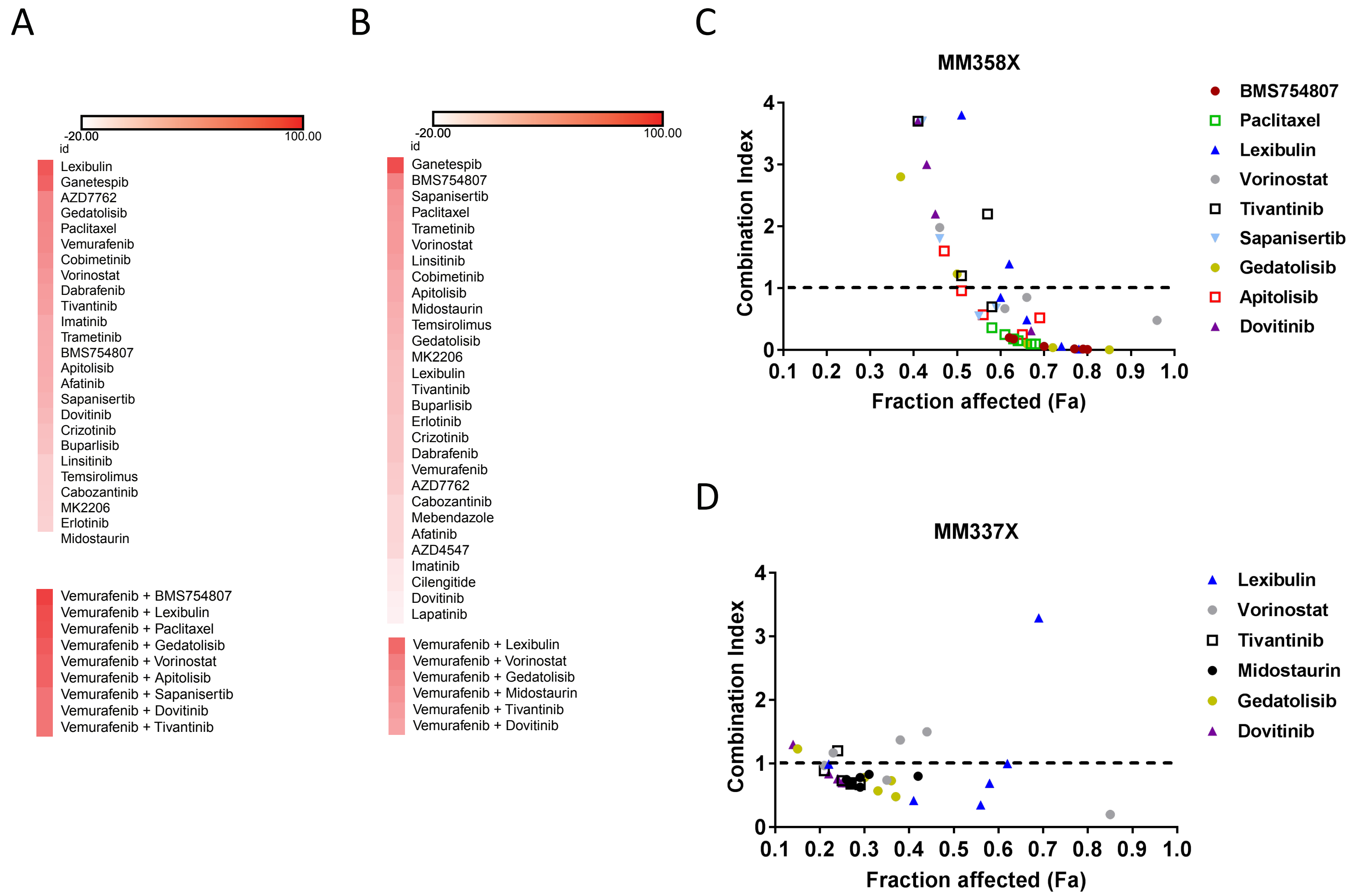


Supplementary Figure 5

| Drug | Cmax μM | PMID | PMCID |
|---|---|---|---|
| Afatinib | 1 | 28364015 | PMC5511563 |
| Apitolisib | 1 | 26787751 | PMC4876928 |
| Axitinib | 0.2 | 28364015 | PMC5511563 |
| AZD4547 | 0.5 | 28070720 | PMC5502072 |
| AZD7762 | 0.5 | 24448638 | PMC4486055 |
| Bosutinib | 0.4 | 28364015 | PMC5511563 |
| Buparlisib | 2 | 24405565 | PMC4317947 |
| Cabozantinib | 1 | 28364015 | PMC5511563 |
| Cilengitide | 50 | 12706360 | N/A |
| Cobimetinib | 1 | 28364015 | PMC5511563 |
| Crizotinib | 1 | 28364015 | PMC5511563 |
| Dabrafenib | 10 | 28364015 | PMC5511563 |
| Dacomitinib | 0.05 | 22249430 | PMC3523469 |
| Dasatinib | 0.3 | 28364015 | PMC5511563 |
| Dinaciclib | 1 | 25217392 | N/A |
| Dovitinib | 1 | 23339124 | N/A |
| Doxorubicin | 15 | 28364015 | PMC5511563 |
| Erlotinib | 2 | 28364015 | PMC5511563 |
| Everolimus | 0.06 | 28364015 | PMC5511563 |
| Ganetespib | 10 | 23530663 | PMC3626541 |
| Gedatolisib | 1 | 27103175 | N/A |
| Imatinib | 7.5 | 28364015 | PMC5511563 |
| Ipatasertib | 1.5 | 27872130 | PMC5463454 |
| Lapatinib | 2 | 28364015 | PMC5511563 |
| Lexibulin | 1 | 20733579 | PMC2938266 |
| Linsitinib | 10 | 25795408 | N/A |
| LY2228820 | 2 | 26581242 | N/A |
| Mebendazole | 1 | 7094986 | N/A |
| Midostaurin | 10 | 20733134 | PMC4135183 |
| MK2206 | 0.4 | 25239610 | PMC4233149 |
| Olaparib | 10 | 28364015 | PMC5511563 |
| Paclitaxel | 5 | 28364015 | PMC5511563 |
| Palbociclib | 0.1 | 28364015 | PMC5511563 |
| PX866 | 0.1 | 22693357 | N/A |
| Regorafenib | 10 | 28364015 | PMC5511563 |
| Sapanisertib | 0.3 | 26800393 | N/A |
| Semagacestat | 0.2 | 18695053 | PMC2682361 |
| Sunitinib | 0.2 | 28364015 | PMC5511563 |
| Temsirolimus | 0.5 | 28364015 | PMC5511563 |
| Tivantinib | 19 | 23413279 | N/A |
| Tozasertib | 4 | 20386909 | PMC3050703 |
| Trametinib | 0.02 | 28364015 | PMC5511563 |
| Veliparib | 10 | 27803064 | N/A |
| Vemurafenib | 50 | 28364015 | PMC5511563 |
| Vismodegib | 35 | 28364015 | PMC5511563 |
| Vorinostat | 10 | 15857402 | PMC6040651 |

Supplementary Table 1

| Case | Age | Gender | Primary Tumor Type | Site of Tissue Biopsy | Therapies | BRAF status |
|---|---|---|---|---|---|---|
| MM300 | 69 | M | Cutaneous (right cheek) | LN | dabrafenib + Ipilimumab | BRAF V600K |
| MM302 | 69 | M | Cutaneous (right foot) | Distant Met | dabrafenib, dabrafenib + ipilimumab | BRAF V600E |
| MM313 | 69 | F | Cutaneous (back) | Distant Met | Naïve | BRAF V600E |
| MM314 | 69 | M | Unknown Primary | LN | Naïve | BRAF V600E |
| MM321 | 79 | M | Unknown Primary | LN | dabrafenib + trametinib | BRAF V600E |
| MM325 | 69 | F | Cutaneous (left leg) | LN | ipilimumab, nivolumab, Ipilimumab + nivolumab, nivolumab | BRAF V600E |
| MM334 | 69 | M | Unknown Primary | Brain | high-dose interferon-alpha, dabrafenib + trametinib + ipilimumab | BRAF V600K |
| MM337 | 49 | F | Cutaneous (left ear) | LN | biochemotherapy, pulse interleukin-2/GM-CSF, vemurafenib, ipilimumab, pembrolizumab, pembrolizumab + dabrafenib, carboplatin + paclitaxel, nivolumab + ipilimumab, nivolumab | BRAF V600E |
| MM354 | 39 | M | Cutaneous (chest) | Distant Met | pembrolizumab, dabrafenib + trametinib, ipilimumab + nivolumab | BRAF V600E |
| MM358 | 59 | M | Cutaneous (back) | Brain | dabrafenib + trametinib | BRAF V600K |

Supplementary Table 2

| GENE | MM300 | MM300X | MM313 | MM313X | MM314 | MM314X | MM325 | MM325X | MM334 | MM334X | MM354 | MM354X1 | MM358 | MM358X |
|---|---|---|---|---|---|---|---|---|---|---|---|---|---|---|
| ABL1 | 0.14 | | | | | | | | | | | | | |
| ABL1 | 0.13 | | | | | | | | | | | | | |
| ABL1 | | | | | | | | | | 0.09 | | | | |
| APC | | | | | 0.50 | 0.68 | | | | | | | | |
| APC | | | 0.21 | | | | | | | | | | | |
| ATM | | | | | | | 0.83 | 0.98 | | | | | | |
| BRAF | | | 0.17 | 0.57 | 0.26 | 0.78 | 0.60 | 0.67 | | | 0.65 | 0.67 | | |
| BRAF | 0.54 | 1.00 | | | | | | | 0.83 | 0.84 | | | 0.77 | 0.61 |
| CTNNB1 | 0.06 | | | | | | | | | | | | | |
| ERBB4 | | 0.27 | | | | | | | | | | | | |
| FBXW7 | | | | 0.09 | | | | | | | | | | |
| FGFR3 | | 0.18 | | | | | | | | | | | | |
| FLT3 | 0.14 | | | | | | | | | | | | | |
| GNA11 | | | | | | | | | | 0.09 | | | | |
| GNAQ | | | | | | | | | | 0.07 | | | | |
| GNAQ | | | 0.06 | | | | | | | | | | | |
| HNF1A | | | | | | | | | | | | 0.05 | 0.09 | |
| HNF1A | | | | | 0.05 | | | | | | | | | |
| HNF1A | | | 0.06 | 0.08 | 0.17 | 0.05 | | | | | | | | |
| KDR | 0.09 | | | | | | | | | | | | | |
| KDR | | | 0.08 | 0.28 | 0.51 | 0.65 | 0.50 | 0.51 | | | | | | |
| KIT | | | 0.05 | | | | | | | | | | | |
| KIT | | | | 0.24 | | | | | | | | | | |
| KIT | | | 0.06 | | | | | | | | | | | |
| MET | 0.11 | | | | | | | | | | | | | |
| NPM1 | | | 0.20 | | | | | | | | | | | |
| NPM1 | | | 0.18 | | | | | | | | | | | |
| NRAS | | | 0.06 | | | | | | | | | | | |
| NRAS | 0.10 | | | | | | | | | | | | | |
| NRAS | 0.26 | | | | | | | | | | | | | |
| PTEN | | | | | | | | | 0.41 | 0.56 | | | | |
| RB1 | 0.05 | | | | | | | | | | | | | |
| RB1 | 0.06 | | | | | | | | | | | | | |
| RB1 | 0.06 | | | | | | | | | | | | | |
| RB1 | | | | | | | | | | | 0.12 | | | |
| SMAD4 | 0.11 | | | | | | | | | | | | | |
| SMARCB1 | | | | | | | | | 0.42 | 0.51 | | | | |
| STK11 | 0.10 | | | | | | | | | | | | | |
| TP53 | 0.54 | 0.97 | | | | | | | | | | | | |
| | | | | | | | | | | | | | | |
| Number of variants (any variant) | 15 | 4 | 10 | 5 | 5 | 4 | 3 | 3 | 3 | 6 | 2 | 2 | 2 | 1 |
| Number of variants (variant ≥ 10%) | 9 | 4 | 4 | 3 | 4 | 3 | 3 | 3 | 3 | 3 | 2 | 1 | 1 | 1 |
| | 3 | 3 | 0 | 2 | 3 | 3 | 3 | 3 | 3 | 3 | 1 | 1 | 1 | 1 |
| | MM300 | MM300X | MM313 | MM313X | MM314 | MM314X | MM325 | MM325X | MM334 | MM334X | MM354 | MM354X1 | MM358 | MM358X |
| Concordance (any variant) | 0.12 | | 0.3 | | 0.8 | | 1.0 | | 0.5 | | 0.3 | | 0.5 | |
| Concordance (variant ≥ 10%) | 0.2 | | 1.0 | | 1.0 | | 1.0 | | 1.0 | | 0.5 | | 1.0 | |
| Concordance (variant ≥ 25%) | 0.5 | | 1.0 | | 1.0 | | 1.0 | | 1.0 | | 1 | | 1 | |
| | | | | | | | | | | | | | | |
| Total Concordance (any variant) | 0.35 | | | | | | | | | | | | | |
| Total Concordance (variant ≥ 10%) | 0.48 | | | | | | | | | | | | | |
| Total Concordance (variant ≥ 25%) | 0.88 | | | | | | | | | | | | | |

Supplementary Table 3

| GENE | MM300X | MM300X1 | MM302X | MM302X1 | MM313X | MM313X1 | MM314X | MM314X1 | MM325X | MM325X1 | MM334X | MM334X1 | MM337X | MM337X1 |
|---|---|---|---|---|---|---|---|---|---|---|---|---|---|---|
| ABL1 | | | | | | | | | | | 0.09 | | | |
| APC | | | | | | | 0.68 | 0.66 | | | | | | |
| ATM | | | | | | | | | 0.98 | 1.00 | | | | |
| BRAF | | | 0.61 | 0.64 | 0.57 | 0.50 | 0.78 | 0.79 | 0.67 | 0.66 | | | 0.24 | 0.23 |
| BRAF | 1.00 | 0.99 | | | | | | | | | 0.84 | 0.87 | | |
| CTNNB1 | | | | | | | | | | | | | 0.25 | 0.25 |
| EGFR | | | | | | 0.12 | | | | | | | | |
| ERBB4 | 0.27 | 0.29 | | | | | | | | | | | | |
| FBXW7 | | | | | 0.09 | | | | | | | | | |
| FGFR3 | 0.18 | | | | | | | | | | | | | |
| GNA11 | | | | | | | | | | | 0.09 | | | |
| GNAQ | | | | | | | | | | | 0.07 | | | |
| HNF1A | | 0.08 | | | 0.08 | | 0.05 | | | 0.05 | | | | |
| KDR | | | 0.52 | 0.50 | 0.28 | 0.54 | 0.65 | 0.63 | 0.51 | 0.49 | | | 0.99 | 1.00 |
| KIT | | | | | 0.24 | 0.40 | | | | | | | | |
| NRAS | | | | | | | | | | | | | 0.23 | 0.25 |
| PTEN | | | | | | | | | | | 0.56 | 0.46 | | |
| RB1 | | | | | | | | | | | | | | 0.09 |
| RB1 | | | | | | | | 0.05 | | | | | | |
| SMARCB1 | | | | | | | | | | | 0.51 | 0.68 | | |
| TP53 | 0.97 | 0.97 | | | | | | | | | | | | |
| | | | | | | | | | | | | | | |
| Number of variants (any variant) | 4 | 4 | 2 | 2 | 5 | 4 | 4 | 4 | 3 | 4 | 6 | 3 | 4 | 5 |
| Number of variants (variant ≥ 10%) | 4 | 3 | 2 | 2 | 3 | 4 | 3 | 3 | 3 | 3 | 3 | 3 | 4 | 4 |
| Number of variants (variant ≥ 25%) | 3 | 3 | 2 | 2 | 2 | 3 | 3 | 3 | 3 | 3 | 3 | 3 | 2 | 3 |
| | MM300 | MM300X | MM302X | MM302X1 | MM313X | MM313X1 | MM314 | MM314X | MM325 | MM325X | MM334 | MM334X | MM337X | MM337X1 |
| Concordance (any variant) | 0.6 | | 1.0 | | 0.5 | | 0.6 | | 0.8 | | 0.5 | | 0.8 | |
| Concordance (variant ≥ 10%) | 0.8 | | 1.0 | | 0.8 | | 1.0 | | 1.0 | | 1.0 | | 1.0 | |
| Concordance (variant ≥ 25%) | 1 | | 1.0 | | 1 | | 1 | | 1 | | 1 | | 0.7 | |
| | | | | | | | | | | | | | | |
| Total Concordance (any variant) | 0.64 | | | | | | | | | | | | | |
| Total Concordance (variant ≥ 10%) | 0.91 | | | | | | | | | | | | | |
| Total Concordance (variant ≥ 25%) | 0.95 | | | | | | | | | | | | | |

Supplementary Table 3

| | MM314X1 | MM337X1 | MM321X2 | MM358X | MM302X | MM334X1 | MM313X | MM325X |
|---|---|---|---|---|---|---|---|---|
| Vemurafenib | 56.2 | 23.0 | 38.1 | 18.4 | 8.5 | 91.8 | 51.8 | 92.7 |
| Dabrafenib | 64.6 | 28.6 | 33.6 | 23.3 | 28.7 | 79.7 | 52.6 | 94.6 |
| Trametinib | 40.2 | 14.7 | 23.2 | 23.8 | 50.5 | 53.0 | 38.4 | 69.9 |
| Cobimetinib | 80.9 | 48.0 | 37.4 | 23.0 | 77.2 | 90.9 | 50.5 | 95.1 |
| Vemurafenib + Cobimetinib | 91.2 | 60.9 | 36.6 | 27.8 | 78.4 | 97.5 | 54.3 | 95.1 |
| Crizotinib | -2.5 | 9.3 | 15.9 | -9.8 | 22.7 | 18.7 | 12.5 | 19.7 |
| Tivantinib | 9.6 | 9.4 | -8.3 | -1.6 | -1.7 | 15.6 | -3.3 | -27.7 |
| Dasatinib | -9.4 | -10.0 | 22.6 | -21.9 | -12.6 | 2.4 | -13.9 | 8.0 |
| Bosutinib | -7.1 | 0.4 | 25.3 | -11.7 | -15.7 | -13.7 | -6.2 | 1.7 |
| Erlotinib | 13.5 | 34.7 | 20.9 | 2.8 | 18.6 | 30.1 | -0.1 | 18.9 |
| Lapatinib | -2.2 | -17.1 | -4.8 | -7.7 | -17.6 | -1.1 | -3.8 | -7.7 |
| Axitinib | 1.5 | 4.1 | 13.6 | -12.8 | -13.0 | 2.9 | -5.1 | 4.7 |
| Regorafenib | 24.5 | 22.5 | 28.2 | 3.2 | 44.3 | 23.0 | 17.2 | 23.7 |
| Sunitinib | 17.9 | 34.8 | 30.9 | 5.0 | -6.6 | 5.4 | 23.3 | 18.4 |
| Imatinib | 0.5 | -9.7 | -8.1 | -6.9 | -28.8 | -3.4 | -8.6 | -17.6 |
| Linsitinib | 2.2 | 40.4 | 17.2 | 21.4 | 77.5 | 34.4 | 2.8 | 40.2 |
| LY2228820 | -2.4 | -10.3 | -0.9 | -6.1 | -6.6 | 6.4 | -11.7 | -20.4 |
| Dacomitinib | -6.0 | -5.2 | 7.6 | -3.5 | -18.7 | -25.3 | -3.2 | -7.0 |
| Temsirolimus | 21.0 | 48.0 | 29.9 | 0.6 | 29.4 | 20.5 | 26.6 | 40.9 |
| Buparlisib | 9.0 | 26.1 | 6.3 | 8.0 | 17.4 | 6.3 | 30.4 | 17.9 |
| Everolimus | 19.5 | 48.2 | 2.7 | 14.0 | 15.0 | -6.2 | 26.7 | 29.0 |
| Ipatasertib | 3.2 | -4.7 | 8.3 | -0.2 | -4.4 | 1.6 | 4.5 | -13.2 |
| MK2206 | -4.7 | -11.1 | 14.7 | -5.9 | -9.0 | -2.2 | 3.1 | -4.8 |
| Sapanisertib | 34.3 | 46.7 | 16.0 | 15.1 | 40.3 | 34.1 | 35.4 | 32.8 |
| PX866 | -12.8 | -0.6 | -2.0 | -1.3 | -18.6 | -9.6 | -1.4 | -1.3 |
| Palbociclib | -3.0 | -11.5 | 12.0 | -11.6 | -4.6 | 5.9 | -5.0 | 6.0 |
| Dinaciclib | 60.6 | 81.1 | 44.9 | 49.9 | 54.1 | 63.7 | 47.9 | 43.4 |
| Doxorubicin | 63.9 | 94.1 | 69.8 | 18.9 | 37.1 | 16.3 | 49.7 | 44.4 |
| Vismodegib | 0.7 | 4.7 | 25.4 | -8.7 | -0.4 | -2.2 | 6.7 | 13.9 |
| Ganetespib | 80.4 | 66.7 | -6.3 | 29.9 | 72.6 | 52.7 | 47.9 | 69.4 |
| Olaparib | 2.6 | 13.1 | 30.0 | -12.0 | -12.9 | -9.9 | -9.7 | 3.0 |
| Veliparib | 7.4 | 5.1 | -1.6 | 0.0 | -5.7 | -1.7 | -6.0 | -19.2 |
| Semagacestat | 26.3 | 21.1 | 11.1 | 14.6 | -3.3 | 24.6 | 19.0 | 24.0 |
| Tozasertib | 18.9 | 36.3 | 23.3 | -2.7 | 32.9 | 7.4 | 14.4 | 17.4 |
| Vorinostat | 74.4 | 58.6 | 72.8 | 28.6 | 31.1 | 27.5 | 67.1 | 52.4 |

Supplementary Table 4

| | MM314X1 | MM337X1 | MM321X2 | MM358X | MM302X | MM334X1 | MM313X | MM325X |
|---|---|---|---|---|---|---|---|---|
| Vemurafenib | 118.3 | 207.9 | 167.2 | 220.2 | 247.1 | 22.1 | 130.1 | 19.7 |
| Dabrafenib | 95.5 | 192.7 | 179.2 | 207.0 | 192.6 | 54.9 | 128.0 | 14.6 |
| Trametinib | 161.5 | 230.4 | 207.4 | 205.7 | 133.6 | 126.9 | 166.3 | 81.3 |
| Cobimetinib | 51.5 | 140.5 | 168.9 | 207.8 | 61.6 | 24.5 | 133.6 | 13.3 |
| Vemurafenib + Cobimetinib | 23.7 | 105.6 | 171.2 | 195.0 | 58.3 | 6.9 | 123.4 | 13.3 |
| Crizotinib | 276.7 | 245.0 | 227.0 | 296.5 | 208.7 | 219.6 | 236.2 | 216.9 |
| Tivantinib | 244.0 | 244.7 | 292.4 | 274.4 | 274.5 | 228.0 | 278.9 | 344.7 |
| Dasatinib | 295.3 | 296.9 | 209.1 | 329.2 | 303.9 | 263.4 | 307.6 | 248.5 |
| Bosutinib | 289.2 | 268.9 | 201.7 | 301.5 | 312.5 | 307.1 | 286.7 | 265.3 |
| Erlotinib | 233.6 | 176.2 | 213.5 | 262.4 | 219.7 | 188.7 | 270.3 | 219.1 |
| Lapatinib | 276.0 | 316.1 | 283.0 | 290.7 | 317.4 | 273.1 | 280.2 | 290.7 |
| Axitinib | 265.9 | 258.9 | 233.4 | 304.6 | 305.0 | 262.2 | 283.9 | 257.3 |
| Regorafenib | 203.9 | 209.2 | 193.9 | 261.4 | 150.4 | 207.8 | 223.5 | 206.1 |
| Sunitinib | 221.6 | 176.0 | 186.7 | 256.6 | 287.8 | 255.4 | 207.2 | 220.2 |
| Imatinib | 268.7 | 296.2 | 292.0 | 288.5 | 347.8 | 279.2 | 293.3 | 317.6 |
| Linsitinib | 264.1 | 160.8 | 223.6 | 212.2 | 60.9 | 177.1 | 262.5 | 161.5 |
| LY2228820 | 276.6 | 297.8 | 272.3 | 286.5 | 287.7 | 252.7 | 301.6 | 325.1 |
| Dacomitinib | 286.1 | 284.0 | 249.4 | 279.4 | 320.6 | 338.2 | 278.7 | 289.0 |
| Temsirolimus | 213.2 | 140.3 | 189.3 | 268.3 | 190.6 | 214.6 | 198.1 | 159.5 |
| Buparlisib | 245.7 | 199.5 | 253.1 | 248.3 | 223.1 | 253.0 | 187.9 | 221.7 |
| Everolimus | 217.4 | 139.9 | 262.8 | 232.1 | 229.5 | 286.8 | 197.8 | 191.8 |
| GDC0068 (Ipatasertib) | 261.4 | 282.6 | 247.6 | 270.5 | 282.0 | 265.6 | 257.9 | 305.7 |
| MK2206 | 282.6 | 299.9 | 230.3 | 285.8 | 294.2 | 275.9 | 261.5 | 283.0 |
| MLN0128 (Sapanisertib) | 177.4 | 143.8 | 226.8 | 229.2 | 161.1 | 177.8 | 174.5 | 181.4 |
| PX866 | 304.5 | 271.6 | 275.5 | 273.4 | 320.1 | 295.8 | 273.7 | 273.5 |
| Palbociclib | 278.1 | 301.0 | 237.7 | 301.3 | 282.3 | 254.1 | 283.5 | 253.8 |
| Dinaciclib | 106.5 | 51.1 | 148.7 | 135.4 | 124.0 | 98.0 | 140.7 | 152.7 |
| Doxorubicin | 97.4 | 16.0 | 81.7 | 219.0 | 169.7 | 226.0 | 135.7 | 150.2 |
| Vismodegib | 268.2 | 257.4 | 201.4 | 293.6 | 271.1 | 276.0 | 252.0 | 232.5 |
| Ganetespib | 52.9 | 89.9 | 286.9 | 189.4 | 74.1 | 127.8 | 140.6 | 82.8 |
| Olaparib | 262.9 | 234.6 | 189.1 | 302.3 | 304.7 | 296.7 | 296.3 | 261.9 |
| Veliparib | 250.1 | 256.1 | 274.3 | 270.1 | 285.4 | 274.7 | 286.1 | 321.9 |
| Semagacestat | 199 | 213.1 | 240 | 230.7 | 279 | 203.7 | 218.6 | 205.1 |
| Tozasertib | 219.1 | 172.1 | 207.2 | 277.3 | 181.1 | 250 | 231.1 | 223 |
| Vorinostat | 69 | 111.9 | 73.33 | 192.7 | 186 | 195.7 | 88.88 | 128.4 |

Supplementary Table 4

| | MM302X Score | MM302X AUC |
|---|---|---|
| Ganetespib | 97.5 | 6.8 |
| BMS-754807 | 81.0 | 51.3 |
| Trametinib | 72.8 | 73.6 |
| Paclitaxel | 71.4 | 77.3 |
| Cobimetinib | 66.3 | 90.9 |
| Lexibulin | 66.1 | 91.4 |
| Sapanisertib | 59.5 | 109.3 |
| Gedatolisib | 51 | 132.4 |
| Vorinostat | 43.1 | 153.6 |
| Midostaurin | 40.3 | 161.2 |
| Tivantinib | 35.2 | 175 |
| Apitolisib | 26 | 199.9 |
| Everolimus | 25.5 | 201.2 |
| Dabrafenib | 24.5 | 203.9 |
| Linsitinib | 17.4 | 222.9 |
| MK2206 | 17.1 | 223.8 |
| Afatinib | 12.7 | 235.7 |
| Buparlisib | 12.3 | 236.9 |
| Dovitinib | 11.9 | 237.8 |
| Temsirolimus | 8.7 | 246.5 |
| AZD7762 | 3.2 | 261.4 |
| Lapatinib | 1.5 | 266 |
| Erlotinib | 1.3 | 266.5 |
| Cilengitide | -0.4 | 271 |
| Cabozantinib | -3.7 | 279.9 |
| XL765 | -4.6 | 282.4 |
| Mebendazole | -6.8 | 288.3 |
| Vemurafenib | -9.5 | 295.6 |
| AZD4547 | -9.7 | 296.1 |
| Imatinib | -20.9 | 326.5 |
| Crizotinib | -21.1 | 326.9 |

Supplementary Table 5

| | MM358X Score | MM358X AUC |
|---|---|---|
| Lexibulin | 59.9 | 108.2 |
| Ganetespib | 54.0 | 124.2 |
| AZD7762 | 37.4 | 168.9 |
| Gedatolisib | 37.3 | 169.2 |
| Paclitaxel | 35.6 | 174 |
| Vemurafenib | 34.9 | 175 |
| Cobimetinib | 32.4 | 182.6 |
| Vorinostat | 28.6 | 192.7 |
| Dabrafenib | 25.9 | 200.1 |
| Tivantinib | 24.6 | 203.6 |
| Everolimus | 23.6 | 206.4 |
| Imatinib | 19.4 | 217.6 |
| Trametinib | 19.0 | 218.7 |
| Apitolisib | 18.0 | 221.5 |
| Afatinib | 17.3 | 223.2 |
| Sapanisertib | 14.9 | 229.9 |
| Dovitinib | 12.4 | 236.5 |
| Crizotinib | 9.4 | 244.5 |
| Buparlisib | 8.5 | 247.1 |
| Linsitinib | 3.2 | 261.3 |
| Temsirolimus | 3.2 | 261.3 |
| Cabozantinib | 2.4 | 263.6 |
| MK2206 | 1.9 | 265 |
| Erlotinib | 1.3 | 266.5 |
| Midostaurin | -27.4 | 343.9 |

Supplementary Table 5

| | MM337X Score | MM337X AUC |
|---|---|---|
| Ganetespib | 65.4 | 93.5 |
| Sapanisertib | 30.8 | 186.8 |
| BMS754807 | 37.8 | 168.0 |
| Paclitaxel | 28.7 | 192.6 |
| Trametinib | 27.7 | 195.3 |
| Vorinostat | 27.4 | 196.1 |
| Linsitinib | 24.6 | 203.6 |
| Cobimetinib | 20.1 | 215.8 |
| Apitolisib | 19.4 | 217.6 |
| Midostaurin | 17.1 | 223.9 |
| XL765 | 16.0 | 226.7 |
| Temsirolimus | 15.1 | 229.2 |
| Gedatolisib | 13.8 | 232.8 |
| MK2206 | 11.2 | 239.7 |
| Lexibulin | 10.7 | 241.0 |
| Tivantinib | 9.4 | 244.7 |
| Buparlisib | 9.1 | 245.5 |
| Erlotinib | 7.3 | 250.4 |
| Crizotinib | 6.8 | 251.6 |
| Dabrafenib | 6.6 | 252.2 |
| Vemurafenib | 4.7 | 257.2 |
| AZD7762 | 4.1 | 258.8 |
| Everolimus | 3.7 | 259.9 |
| Cabozantinib | -0.6 | 271.6 |
| Mebendazole | -0.7 | 271.9 |
| Afatinib | -0.8 | 272.1 |
| AZD4547 | -1.0 | 272.7 |
| Imatinib | -8.7 | 293.4 |
| Cilengitide | -9.1 | 294.6 |
| Dovitinib | -11.6 | 301.3 |
| Lapatinib | -12.4 | 303.4 |

Supplementary Table 5

MM302X

| Drug | Score | AUC | AUC CL | Drug combination | Score | AUC | AUC CL |
|---|---|---|---|---|---|---|---|
| BMS754807 | 81.0 | 51.3 | 44.52 to 58.12 | Vemurafenib + BMS754807 | 86.01 | 37.78 | 34.07 to 41.5 |
| Paclitaxel | 71.4 | 77.3 | 67.1 to 87.5 | Vemurafenib + Paclitaxel | 83.9 | 43.5 | 41.2 to 45.8 |
| Lexibulin | 66.1 | 91.44 | 80.07 to 102.8 | Vemurafenib + Lexibulin | 81.4 | 50.15 | 43.7 to 56.6 |
| Vorinostat | 43.1 | 153.6 | 127.6 to 179.5 | Vemurafenib + Vorinostat | 73.6 | 71.37 | 43.3 to 99.4 |
| Tivantinib | 35.2 | 175 | 145.3 to 204.8 | Vemurafenib + Tivantinib | 68.9 | 98.6 | 88.0 to 109.2 |
| Midostaurin | 40.3 | 161.2 | 148.8 to 173.6 | Vemurafenib + Midostaurin | 68.5 | 92.4 | 80.3 to 104.5 |
| Sapanisertib | 59.5 | 109.3 | 101.7 to 116.9 | Vemurafenib + Sapanisertib | 65.8 | 84.1 | 72.9 to 95.2 |
| Gedatolisib | 51.0 | 132.4 | 122 to 142.8 | Vemurafenib + Gedatolisib | 63.5 | 85.1 | 76.6 to 93.7 |
| Apitolisib | 26.0 | 199.9 | 186.1 to 213.8 | Vemurafenib + Apitolisib | 58.0 | 113.5 | 100.6 to 126.4 |
| Dovitinib | 11.9 | 237.8 | 208.9 to 266.7 | Vemurafenib + Dovitinib | 53.2 | 126.3 | 106.7 to 145.8 |

MM358X

| Drug | Score | AUC | AUC CL | Drug combination | Score | AUC | AUC CL |
|---|---|---|---|---|---|---|---|
| BMS754807 | 36.2 | 172.2 | 153.9 to 190.5 | Vemurafenib + BMS754807 | 71.9 | 75.9 | 70.17 to 81.58 |
| Paclitaxel | 35.6 | 174 | 154.3 to 193.7 | Vemurafenib + Paclitaxel | 63.56 | 98.38 | 85.5 to 111.3 |
| Lexibulin | 59.9 | 108.2 | 93.9 to 122.5 | Vemurafenib + Lexibulin | 65.59 | 92.9 | 71.93 to 113.9 |
| Vorinostat | 28.6 | 192.7 | 176 to 209.3 | Vemurafenib + Vorinostat | 53.89 | 124.5 | 106.1 to 142.9 |
| Tivantinib | 24.6 | 203.6 | 164.7 to 242.5 | Vemurafenib + Tivantinib | 44.52 | 149.8 | 139.2 to 160.5 |
| Sapanisertib | 14.9 | 229.9 | 183.4 to 276.3 | Vemurafenib + Sapanisertib | 45.26 | 147.8 | 135.1 to 160.4 |
| Gedatolisib | 37.3 | 169.2 | 146.2 to 192.1 | Vemurafenib + Gedatolisib | 58.11 | 113.1 | 104.4 to 121.7 |
| Apitolisib | 18.0 | 221.5 | 184.1 to 259 | Vemurafenib + Apitolisib | 53.85 | 124.6 | 112.5 to 136.7 |
| Dovitinib | 12.4 | 236.5 | 217.5 to 255.5 | Vemurafenib + Dovitinib | 45.22 | 147.9 | 134.8 to 161.1 |

MM337X

| Drug | Score | AUC | AUC CL | Drug combination | Score | AUC | AUC CL |
|---|---|---|---|---|---|---|---|
| Lexibulin | 10.74 | 241 | 220.2 to 261.8 | Vemurafenib + Lexibulin | 51.2 | 131.7 | 127 to 136.5 |
| Vorinostat | 27.37 | 196.1 | 176.3 to 215.8 | Vemurafenib + Vorinostat | 39.9 | 162.4 | 152.6 to 172.2 |
| Tivantinib | 9.37 | 244.7 | 222.7 to 266.6 | Vemurafenib + Tivantinib | 25.8 | 200.3 | 186.5 to 214.1 |
| Midostaurin | 17.07 | 223.9 | 205.8 to 241.9 | Vemurafenib + Midostaurin | 30.4 | 188 | 175.9 to 200 |
| Gedatolisib | 13.78 | 232.8 | 219.1 to 246.4 | Vemurafenib + Gedatolisib | 34.5 | 176.8 | 162.3 to 191.3 |
| Dovitinib | -11.59 | 301.3 | 285 to 317.6 | Vemurafenib + Dovitinib | 22.8 | 208.5 | 197.4 to 219.7 |

Supplementary Table 5
